# Vera Cordeiro: addressing diseases of poverty

**DOI:** 10.2471/BLT.22.031022

**Published:** 2022-10-01

## Abstract

Vera Cordeiro talks to Andréia Azevedo Soares about creating a community-level strategy to tackle the interlinked challenges of poverty and health.

*Q: What led you to your focus on the social determinants of health?*

A: From the start of my career in medicine I was always interested in the people behind the diseases I was being asked to treat. I worked for 20 years at the Hospital da Lagoa, a public hospital in Rio which serves some of the city’s poorest communities, including those in Rocinha, Rio’s largest favela where around 180 000 live on less than a square mile of land. So, it was hard not to notice that many of the people who came in were impacted by their living conditions. Initially, I was more focused on psychosomatic conditions. This was an interest I had developed studying under Julio de Mello Filho and Abraham Josek Eksterman at university, both influential thinkers in the field. In 1979, with the help of my colleagues, I founded a psychosomatic medicine unit at the hospital.

My focus on social determinants really developed when I became head of the psychosomatic department of the paediatric unit in 1980. I began to notice that many of the children were succumbing to a cycle of hospitalization, discharge, relapse and death. They would come in, we would treat them and then they would go home, get sick again and die. I remember one case that really brought the problem home to me. A child arrived at the service with pneumonia and, after a conversation with the mother, we learned that the house where they lived had a damaged roof. Water was coming in every time it rained. An antibiotic was prescribed for the pneumonia, but I knew that what we really needed to treat was the hole in the roof along with all the other problems this family faced. The pneumonia was just the tip of the iceberg. Underneath it, hidden from us in the normal practice of medicine, was a whole host of socioeconomic problems: poor housing and malnutrition, but also unemployment, alcoholism, addiction, domestic violence. I became convinced that we needed to address poverty itself as a disease.

*Q: You set up the Associação Saúde Criança Renascer (now the Dara Institute) in 1991. Can you explain how that came about?*

A: I reached a point where I felt that I had to do something beyond the walls of the hospital. The anguish grew and grew until I finally said, "enough is enough!” I started using my free afternoons to receive patients in a park near the public hospital. Along with a group of volunteers, I would listen to the families coming into the hospital and tried to understand the conditions in which they lived, and what their basic needs were. We also talked to them about any skills or abilities they might have and started putting them on the road to employment. For example, if a mother reported having a knack for hairstyling, we would pay for a hairdressing course for her.

*Q: How did you raise the funds required to help these families?*

A: In the very beginning, we asked for contributions from friends and colleagues and managed to bring together a thousand supporters. We also did solidarity sales to raise money. Then, in 1992, I became a fellow of Ashoka and that made it much easier to get funding.

“We needed to address poverty itself as a disease.”

*Q: Can you explain?*

A: Ashoka is an international organization that was founded by Bill Drayton in 1980 and it supports social entrepreneurs around the world. The organization is backed by McKinsey & Company (a management consultant firm) who create strategic missions and conduct evaluations of social impact studies and also support Ashoka fellows with personal consultations that help them launch and scale initiatives. Ashoka liked our focus on economic empowerment and the social determinants of health and started sending us US$ 650 a month. But their support didn’t stop there; they also made me and my work more visible. In 1997, as an Ashok fellow, I was invited to give a lecture as a guest in Bahia, Brazil. When I finished speaking, I received a standing ovation and was approached by some people who set up a meeting the next day with Frederico Oliveira, a director in McKinsey's São Paulo office. He helped me design a fundraising plan and prepare grant applications.

*Q: Did you go forward with those?*

A: As soon as I could. In 1998, I went to the National Development Bank of Brazil and asked for a non-refundable grant of 500 000 Brazilian reals (roughly US$ 500 000 at that time) and the request was approved. This was the first significant funding we received, and it made a huge difference, allowing us to hire social workers, psychologists and administrative staff. We have continued to grow since then and today have around 50 employees, supported by 100 volunteers. McKinsey also offered four years of free consultancy to support our work in various areas, from finance to communications. That support was invaluable to us since we were really growing fast and hospital professionals from other states were coming to us to learn about our methods.

*Q: Can you describe those methods?*

A: We start with a careful selection process. We go into hospitals, selected schools and social centres and work closely with staff and families to identify eligible families. Each potential candidate is reviewed in depth to identify the most critical problem the family has. If there is an alcohol problem in the family, for example, food and medicines will not be enough; counselling will be required too. We also visit the family’s home and community, so that we can verify that all the information provided is accurate. This learning process, this listening and observing, is what makes the method so effective. Each poverty story is different from the next. In one case, there is a depressed mother, in the other, we have an addicted father and in another, the house is falling apart and in the middle of it all, there is a child. Our response is to work with the family to design an integrated, personalized family action plan which we then execute in a multidisciplinary way. For example, the architect who will design the roof replacement is in touch with the social worker who is also working closely with doctors.

*Q: What kind of support do you provide exactly?*

A: As needed, we offer medication, psycho-therapeutical support, HIV information, access to chemotherapy, help with drug and alcohol abuse, referrals to self-help groups. To ensure health-care access we also offer transportation vouchers and support with organizing work schedules so that a parent may attend to the health care needs of the child. But beyond all that, we seek to empower the patients and their families. So, during our initial engagement, we identify any talents, skills or capacities that exist in the family and the community around them. We want people to leave Dara as masters of their own destiny, which of course puts a lot of responsibility on them.

“The socioeconomic situation will be very challenging in the coming years.”

We explain to the families that they will receive a great deal of personalized assistance regarding their basic needs, housing, family income, education and citizenship but will also have to participate and make their contribution. So together with the family, we set goals such as enrolling all the children in school, making sure they are up to date with vaccinations, attending the vocational course chosen. All of these things are part of an agreement that is made between the head of the household and the Dara Institute. We organize monthly meetings so that we can see how things are evolving. Where families are not following through with what they agreed to, they are asked to make way for another family.

*Q: At what point can the families stand on their own feet?*

A: The process usually takes two years, but as I say the situations are all different. There are families with children affected by cancer or sickle cell anaemia who are discharged because the chronic disease is controlled, well monitored by a hospital and the mothers are able to accompany them. If it is an acute illness, it must be cured. All children must be in school. The housing in which they live must be minimally acceptable. There are well-defined criteria in the family action plan. Once the objectives have been achieved, the family is discharged to make room for a new vulnerable one.

*Q: Dara Institute works closely with private sector partners. What demands does that partnership impose?*

A: In fact, several national and international foundations also give us financial and technical support and we ensure full accountability, for example, by conducting and publishing annual audits. The private sector trusts us because we have built a relationship based on transparency, accountability and results. Of course, those things are also vitally important if one of your main aims is to see the method replicated. People want to know what things cost, what the implementation challenges are, how effective the interventions are. We have so far served around 85 000 people across Brazil and inspired similar programmes in the United States of America, and in countries in Africa, Asia and Latin America.

*Q: You clearly have reasons for satisfaction. Can you share any of your concerns?*

A: For Dara, I am not concerned, but I am concerned about my country – concerned about the return of famine in the wake of the pandemic and considering the instability in the global food system and the economy. We recently engaged in a three-month consultation to prepare for future economic shocks and realized that the knowledge we have accumulated over three decades is a treasure that needed to be shared. So, we are building a knowledge hub that can be accessed in Portuguese and English from anywhere. We also know that, alone, we will not solve the problem of structural poverty in Brazil and will need to work with partners – for example, through the 8 000 Social Centres in Brazil known as CRAS (Centro de Referencia de Assistencia Social) which have been set up by the government primarily in areas of greater social vulnerability to provide social assistance services. We know that the socioeconomic situation will be very challenging in the coming years, but I believe we have the capacity and the plans we need to survive the storm.

